# Case Report: HER2-positive vulvar Paget disease achieving long-term control with trastuzumab-based systemic therapy plus radiotherapy

**DOI:** 10.3389/fmed.2026.1740493

**Published:** 2026-01-26

**Authors:** Xiaohui Xie, Qinyang Chen, Jian Zhang, Xiaodong Peng

**Affiliations:** 1 West China School of Medicine, Sichuan University Affiliated Chengdu Second People’s Hospital, Chengdu Second People’s Hospital, Sichuan University, Chengdu, Sichuan, China; 2 The Academy of Chinese Health Risks, West China Hospital, Sichuan University, Chengdu, Sichuan, China

**Keywords:** chemoradiotherapy, HER2-positive, HER2-targeted therapy, trastuzumab, vulvar Paget’s disease

## Abstract

**Purpose:**

Vulvar Paget’s disease with secondary invasive adenocarcinoma can be challenging to manage, particularly in inoperable or recurrent situations. We report this rare case to raise clinical awareness, accumulate diagnostic and treatment experience, and offer guidance for managing similar cases in the future.

**Methods:**

We present a 79-year-old woman with large invasive vulvar Paget’s disease (HER2 3+).

**Results:**

As surgery was not feasible, she received trastuzumab plus capecitabine, achieving marked tumor shrinkage. Nine months later, local recurrence with bone metastasis was treated with the same regimen followed by sequential radiotherapy (60 Gy), resulting in partial remission. With trastuzumab maintenance, disease control has been sustained for 17 months.

**Conclusion:**

HER2-targeted therapy combined with chemotherapy and radiotherapy is a promising treatment strategy for HER2-positive, inoperable or recurrent VPD.

## Introduction

Invasive vulvar Paget’s disease (VPD) is an uncommon malignancy, with an incidence of 0.5 cases per 100,000 person-years in the general population (1). Its scarcity has left clinicians with limited experience and, consequently, no evidence-based management guidelines. A definitive diagnosis requires a pathological biopsy. Between 8% and 41% of primary intraepithelial Paget’s disease progresses to invasive disease (2); upon a diagnosis of VPD, a comprehensive systemic work-up for synchronous or metachronous malignancies is mandatory, with particular attention to the vulva, vagina, gastrointestinal tract, and urinary system (3). This case report describes a patient with Invasive VPD who achieved partial remission after targeted therapy combined with chemoradiation.

## Case presentation

**FIGURE 1:**
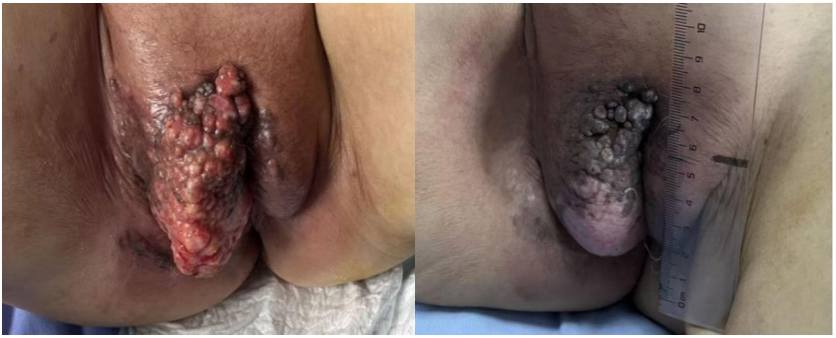
The pre-treatment vulvar mass had a cauliflower-like exophytic appearance with an irregular, nodular surface (left). The post-treatment vulvar mass has significantly shrunk, with a reduction in ulceration and bleeding (right).

**FIGURE 2:**
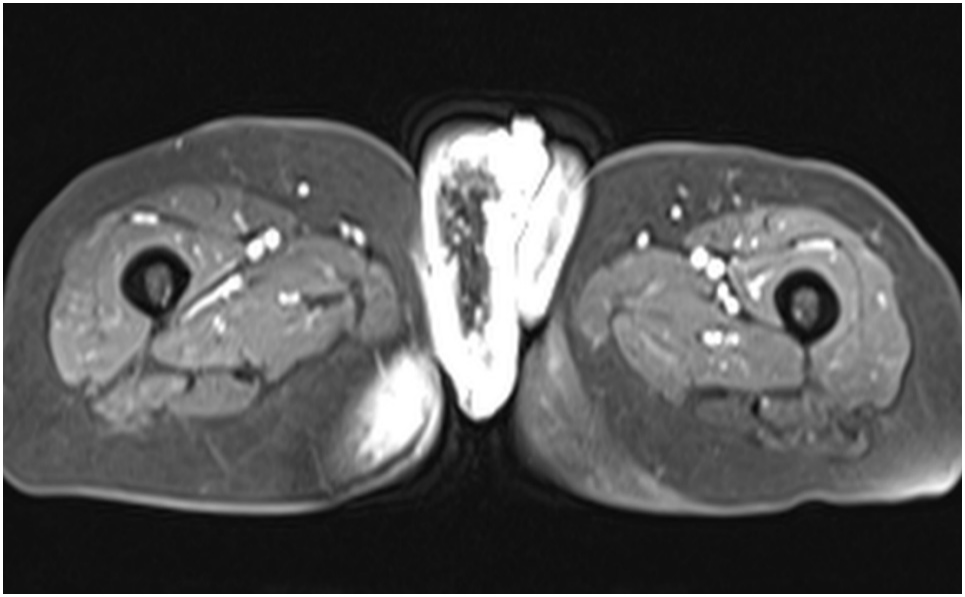
Pelvic MRI showed significant thickening of the vulvar mass and multiple enlarged surrounding lymph nodes.

A 79-year-old female presented with a vulvar mass of unknown cause, accompanied by itching and pain. She had no changes in bowel habits, urinary symptoms, or hematuria. Over the past year, the mass gradually enlarged, with recurrent exudation of bloody fluid from its revealed marked, lobulated thickening surface, and self-applied topical treatments were ineffective (Figure 1). Pelvic MRI of the vulvar skin forming a dominant right-sided mass, accompanied by extensive stranding in the adjacent subcutaneous fat and right gluteal region, together with bilateral enlarged iliac and inguinal lymph nodes (Figure 2). Since the onset of the disease, the patient’s overall condition had remained stable, with good mood, appetite, and sleep. Bowel and bladder functions were normal; however, she had experienced a 12-pound weight loss in the last 3 months.

**FIGURE 3:**
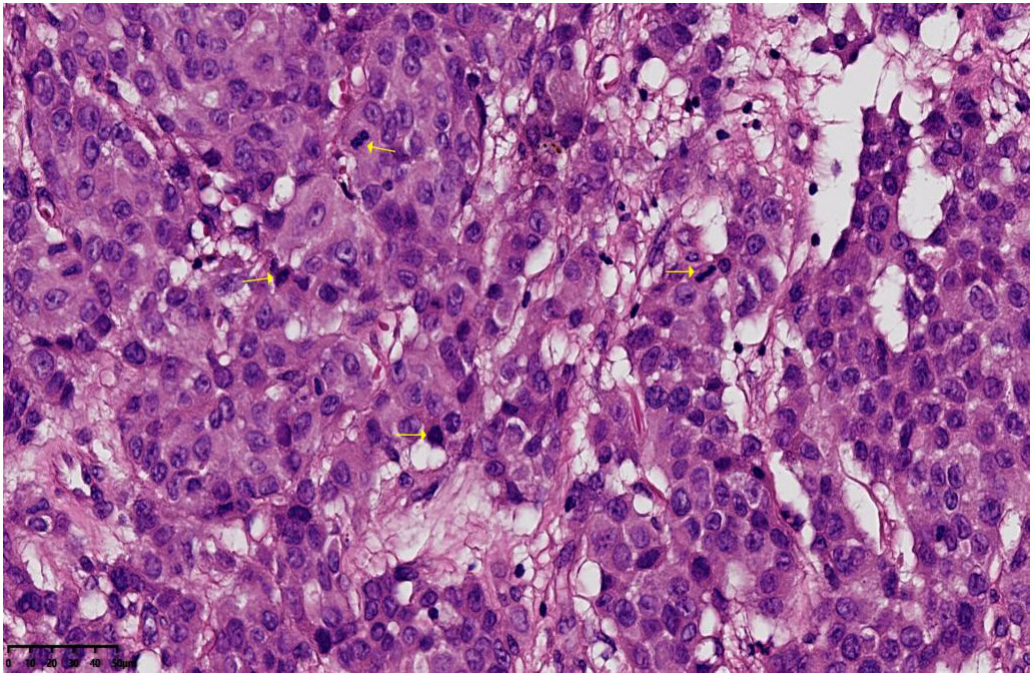
The submitted vulvar tissue shows diffusely distributed tumor cells within multiple foci of the epidermis. The cells are arranged in nests and linear patterns, with eosinophilic cytoplasm. Some cells are rich in mucus and exhibit signet-ring morphology. The nuclei are round to oval, irregular in contour, with thick nuclear membranes, prominent nucleoli, and coarse chromatin. Mitotic figures are easily seen (indicated by yellow arrows).

Biopsy revealed poorly differentiated invasive adenocarcinoma with focal signet-ring features. Immunohistochemistry showed: PCK (diffuse strong ), CK7 (diffuse strong+), GATA3 (diffuse strong+), CK20 (−), CDX-2 (−), P63 (−), CK5/6 (−), GCDFP-15 (−), PAX-8 (−), TTF-1 (−), Napsin A (−), WT1 (−), Calretinin (−), INI-1 (nuclear+), MLH1 (+), MSH6 (+), MSH2 (+), PMS2 (+), ER (+, weak to moderate, approximately 30%), PR (−), Her-2 (3+), E-C (membranous+), S100 (−), Ki-67 (+, approximately 40%), PAS and AB staining: Mucus is present in some tumor cells (Figure 3). Pathological diagnosis: Invasive adenocarcinoma identified in the biopsy tissue, showing poor differentiation with some components exhibiting signet-ring cell morphology. Based on the location and phenotype, primary vulvar tumors should be considered first, including: (1) Vulvar mammary-type adenocarcinoma; (2) Extramammary Paget’s disease with invasive carcinoma. The patient also underwent breast ultrasound, thoracoabdominal pelvic CT, and pelvic MRI, all of which revealed no tumor involvement in adjacent organs, including the rectum and bladder. Given the patient’s advanced age and the high anesthetic risk assessed by the anesthesia department, further colonoscopy was not performed. Taken together, the vulvar location and immunoprofile support the diagnosis of primary extramammary Paget disease with secondary invasive adenocarcinoma.

Because the lesion was bulky and deeply infiltrative, gynecologic evaluation determined that primary resection was infeasible. Given the patient’s advanced age and intolerance to intensive systemic therapy, we elected a well-tolerated regimen of trastuzumab plus capecitabine—an oral agent with proven activity against vulvar Paget’s disease, minimal myelosuppression, mild gastrointestinal toxicity, and excellent adherence. Local radiotherapy will be added once tumor control is achieved. During the treatment, efficacy assessments were conducted after every two treatment cycles. After two cycles, the evaluation showed partial response (PR). After four cycles, PR was maintained, and subsequent examinations revealed no evidence of metastasis to adjacent organs (Figure 1). The treatment showed significant effectiveness. However, limited personal finances prompted the patient to defer further treatment.

**FIGURE 4:**
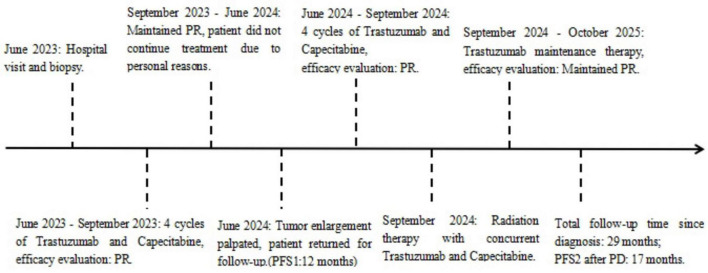
Patient’s diagnosis and treatment flowchart.

Nine months later, the local tumors increased in both number and size, accompanied by multiple subcutaneous nodules in the perineum and lower abdomen, some of which had ulcerated. Pelvic MRI revealed a subtle nodular thickening at the primary vulvar tumor site, accompanied by edema and exudation in the soft tissues of the vulva, buttocks, proximal thigh, and inner pelvic wall. A new small patchy signal was observed in the right iliac bone, suggesting bone metastasis. Thoracoabdominal pelvic CT showed no new masses in the breasts, rectum, bladder, or other adjacent organs. As the patient had responded well to first-line therapy and the treatment-free interval exceeded 6 months, trastuzumab plus chemotherapy was re-initiated for four cycles, resulting in a partial response. To consolidate control, intensity-modulated radiotherapy (60Gy/30f/2.0Gy) was delivered to the vulva and bilateral gluteal lesions, with concurrent oral capecitabine. After completing chemoradiotherapy, the patient was maintained on trastuzumab monotherapy. Due to personal reasons, there was a brief interruption in the first-line treatment, with a progression-free survival (PFS1) of 12 months. After progressive disease (PD), the patient underwent re-treatment, achieving a PFS2 of 17 months. The patient is still on maintenance therapy, with no disease progression to date (Figure 4).

## Discussion

For primary extramammary Paget disease (EMPD) with superficial lesions, no invasive components, and no underlying cancer, the 5-year survival rate can reach 98%–100%, indicating a generally favorable prognosis. However, once metastasis occurs, the survival rate drops sharply to around 52.2%, and the prognosis worsens significantly (4). Surgery remains the cornerstone of management for vulvar Paget disease; standard options range from wide local excision to modified radical vulvectomy and classic radical vulvectomy. For patients who are not suitable for surgery or have positive surgical margins, several alternative non-surgical treatments can be considered, such as topical imiquimod (IMQ) cream, photodynamic therapy, radiation therapy, and systemic chemotherapy (5). After a comprehensive evaluation, we chose local radiotherapy. A retrospective study of 41 primary EMPD patients showed a median dose of 60 Gy, with a 5-year local control rate of 82% and no grade ≥ 3 toxicities. This suggests that radiotherapy is safe and effectively reduces the risk of recurrence (6).

Extramammary Paget disease shares several key targets with breast cancer, such as steroid receptors, the PIK3CA pathway, and HER2 (7). Approximately 40% of invasive VPD cases exhibit HER2 amplification (8). Currently, anti-HER-2 targeted therapy confers clear benefits in primary and metastatic VPD, particularly in the invasive subtype with distant spread (9). Additionally, trastuzumab combined with taxane has been confirmed as an effective treatment regimen in several case series (10). The patient declined intravenous chemotherapy. After a multidisciplinary discussion, trastuzumab combined with capecitabine was chosen as the first-line treatment. The patient quickly achieved PR, with a sustained remission period. Upon local recurrence, the same treatment regimen was re-administered with radiotherapy, leading to another PR and significant improvement in the patient’s quality of life. As anti-HER2 therapies enter the era of advancements in precision medicine, the field of breast cancer has already seen breakthroughs with antibody-drug conjugates (ADCs), such as T-DM1 and T-DXd (11, 12). These novel ADCs deliver cytotoxic payloads with precision through monoclonal antibodies, significantly prolonging survival in HER2-positive breast cancer patients and showing early promise in other solid tumors (13). Building on this success, the future use of ADCs or bispecific antibodies in HER2-positive extramammary Paget’s disease may overcome the limitations of traditional treatments, offering patients deeper, longer-lasting remissions and better prognoses.

## Conclusion

HER2-targeted therapy (trastuzumab) combined with chemotherapy and/or radiation therapy may offer benefits for HER2-positive, inoperable, or recurrent VPD. However, large-scale clinical studies are needed to further explore and validate these findings.

## Patient consent

Written informed consent was obtained from the patient for publication of this case report and the accompanying clinical image. Identifying details have been omitted to protect patient confidentiality.

## Data Availability

The original contributions presented in this study are included in this article/supplementary material, further inquiries can be directed to the corresponding author.
